# The correct prednisone starting dose in polymyalgia rheumatica is related to body weight but not to disease severity

**DOI:** 10.1186/1471-2474-12-94

**Published:** 2011-05-14

**Authors:** Marco A Cimmino, Massimiliano Parodi, Carlomaurizio Montecucco, Roberto Caporali

**Affiliations:** 1 Clinica Reumatologica, Dipartimento di Medicina Interna, Università di Genova, Viale Benedetto XV, 6, 16132 Genova, Italy; 2 IRCCS Policlinico S.Matteo, Pavia, Italy

**Keywords:** polymyalgia rheumatica, prednisone, glucocorticoid, ultrasonography

## Abstract

**Background:**

the mainstay of treatment of polymyalgia rheumatica (PMR) is oral glucocorticoids, but randomized controlled trials of treatment are lacking. As a result, there is no evidence from controlled studies on the efficacy of different initial doses or glucocorticoid tapering. The aim of this study is to test if 12.5 mg prednisone/day is an adequate starting dose in PMR and to evaluate clinical predictors of drug response.

**Methods:**

60 consecutive PMR patients were treated with a starting dose of 12,5 mg/day prednisone. Clinical, laboratory, and, in a subset of 25 patients, ultrasonographic features were recorded as possible predictors of response to prednisone. Remission was defined as disappearance of at least 75% of the signs and symptoms of PMR and normalization of ESR and CRP within the first month, a scenario allowing steroid tapering.

**Results:**

47/60 (78.3%) patients responded to 12.5 mg of prednisone after a mean interval of 6.6 ± 5.2 days. In univariate analysis, body weight and gender discriminated the two groups. In multivariate analysis, the only factor predicting a good response was low weight (p = 0.004); the higher response rate observed in women was explained by their lower weight. The mean prednisone dose per kg in the responders was 0.19 ± 0.03 mg in comparison with 0.16 ± 0.03 mg for non responders (p = 0.007).

**Conclusions:**

12.5 mg prednisone is a sufficient starting dose in ¾ of PMR patients. The main factor driving response to prednisone in PMR was weight, a finding that could help in the clinical care of PMR patients and in designing prospective studies of treatment.

**Trial Registration:**

ClinicalTrials.gov: NCT01169597

## Background

Polymyalgia rheumatica (PMR) is a common inflammatory condition affecting elderly people and involving the girdles [1]. The mainstay of treatment is oral glucocorticoids (GC), with the recent BSR-BHPR guidelines suggesting an initial prednisone dose comprised between 15 and 20 mg as appropriate [2]. However, probably because of the dramatic response of PMR to GC, randomized controlled trials of treatment are lacking. As a result, there is no evidence from controlled studies on the efficacy of different initial doses or drug tapering. The only controlled study suggests that initial prednisone doses ≤ 10 mg is associated with high incidence of recurrences, whereas doses ≥ 20 mg are associated with considerable side effects [3]. Severe steroid toxicity is frequent, occurring in 65% of the patients, and is associated with duration of treatment and cumulative GC dosage [4]. Up to 55% of PMR patients have relapsing disease and require long-term steroid treatment. In one descriptive study, the initial dose was linked to treatment duration and cumulative dosage, for low initial doses were associated with low subsequent maintenance doses [5]. However, most studies are observational and their results could be biased by confounding by indication, i.e. more severe patients being likely to receive higher doses of GC. Identifying the correct starting dose of prednisone for PMR patients could contribute to avoid overtreatment and to reduce the occurrence of side effects.

The aims of this study are (a) to test if 12.5 mg prednisone daily is an adequate starting dose in PMR and (b) to evaluate the factors that could predict a positive response to this initial dose.

## Methods

Sixty consecutive patients with PMR, diagnosed according to the criteria of Bird et al. [6] and visited in two rheumatological tertiary referral centers (Universities of Genova and Pavia, Italy), were considered. Enrolment lasted one year and follow-up lasted one month. An informal chart review was done after 6 month from enrolment to assess the rate of exacerbations. There were 25 men and 35 women; mean age was 71.4 ± 7.2 years. The study protocol was approved by the relevant ethical committees. After written informed consent was obtained, the following demographic and disease characteristics were evaluated: age, sex, body weight measured at the time of the first visit, duration of disease, presence of fatigue, fever, and weight loss, duration of morning stiffness, presence of carpal tunnel syndrome, RS3PE, peripheral arthritis or tenosynovitis. Standardized clinical examination included the following: a) tenderness on palpation of bicipital tendon root, coracoid, lesser and greater tuberosities, and posterior cuff; b) pain worsened by passive and active mobilization, and limitation of motion of the shoulder; c) pain in the groin worsened by passive and active movements and associated with positive Fabere's test, suggesting coxofemoral synovitis; d) aching on the lateral aspect of the hip and thigh, increased by external rotation and abduction and localized tenderness on palpation over the greater trochanter, suggesting trochanteric bursitis; e) tenderness aggravated by extension and relieved by flexion of the hip, suggesting ileo-psoas bursitis; f) pain over the ischions aggravated by sitting and lying, associated with tenderness on palpation over the ischial tuberosities, suggesting ischio-gluteal bursitis; g) paravertebral tenderness and limitation of movement in the lumbar and cervical spine. The two clinical assessors involved in the study (MAC and RC) participated in two training sessions to standardize clinical examination. Patients were instructed to subjectively rate the intensity of fatigue and of the pain experienced during the visit on a scale between 0 and 10. Laboratory and imaging investigations included erythrocyte sedimentation rate (ESR), C-reactive protein (CRP), IgM rheumatoid factor (RF) and ultrasonography (US) of the shoulders. This last examination was performed only in a subset of 25 patients. It included the evaluation of gleno-humeral synovitis (hypoechoic or anechoic effusion larger than 2 mm when measured with a posterior approach and arm in external rotation, or larger than 3.8 mm when measured in the axillary recess between bone and capsule), long head biceps tenosynovitis (hypoechoic or anechoic effusion of the tendon's sheath with diameter larger than 1.4 mm), and subacromial/subdeltoid bursitis (hypoechoic or anechoic effusion with largest sagittal diameter larger than 2 mm). All examinations were performed at the time of the first visit; clinical examination and parameters of inflammation were evaluated also at 1 and 4 weeks. All the patients were treated with 12.5 mg of prednisone daily in the early morning. For the purpose of the study, patients with clinical and laboratory remission of PMR with the above reported dose of prednisone, were considered responders. Remission was defined as at least a 70% global improvement of the signs and symptoms of PMR and normalization of ESR and CRP within the first month, allowing steroid tapering. Non-responders were patients who did not reach remission and, as a consequence, needed an increase in prednisone dosage in the first month of treatment. The interval between treatment initiation and response to it was recorded. The patients were instructed to record on a diary their clinical status and the exact day in which remission was achieved.

Means were compared by the Student's t test or by one way analysis of variance if their distribution was normal and by the Kruskall Wallis test when it was non parametrical. Frequencies were compared by Fischer's exact test. Changes in ESR and CRP during the study period were evaluated between subjects and within subjects by repeated measures ANOVA. A multiple regression model was also used with response to treatment as dependent variable. All the calculations were performed using Medcalc^® ^version 9.6.4.0 (Belgium) as statistical software.

## Results

**Figure 1:**
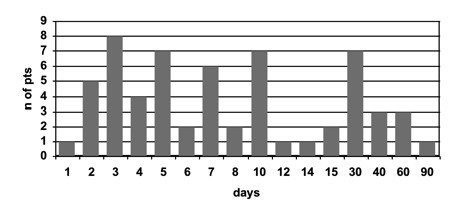
**Interval between initiation of treatment and clinical remission**.

In the 60 consecutive patients with PMR studied, the disease was newly diagnosed and had not been treated with steroids before. The median interval between disease onset and diagnosis was 90 days (range 18-720 days). None of the patients had signs or symptoms suggestive of temporal arteritis at the time of diagnosis or during the observation period. 47/60 (78.3%) patients responded to 12.5 mg of prednisone within one month after beginning therapy. In the responders, the mean interval between initiation of treatment and clinical remission was 6.6 ± 5.2 days (range 1-30 days) (figure 1). The univariate analysis comparison between responders and non-responders is reported in table 1. Only body weight and gender discriminated the two groups of patients. In multivariate analysis, the only factor predicting a good response was a low weight (p = 0.004). The higher response rate observed in women was explained by their lower weight (63.2 ± 7.9 kg vs. 79.2 ± 12.4 kg, p < 0.001). The mean prednisone dose per kg in the responders was 0.19 ± 0.03 mg in comparison with 0.16 ± 0.03 mg for non-responders (p = 0.007). None of the features investigated by physical examination could differentiate responders from non-responders (data not shown). US examination was performed in 21 responders and 4 non-responders. At US, the frequency of gleno-humeral synovitis (p = 0.71), long head biceps tenosynovitis (p = 0.36), and subacromial/subdeltoid bursitis (p = 0.91) was not different in the 2 groups of patients. This was the case also when bilateral involvement of the US pattern was considered.

**Table 1:** Comparison of demographic, clinical, and laboratory results in PMR patients with or without response to 12.5 mg daily of prednisone

FEATURE	RESPONDERS	NON RESPONDERS	p
Number	47 (78.3%)	13 (21.7%)	

Gender (women/men)	31/16	4/9	0.05

Age (years)	71.3 ± 7.1	71.5 ± 7.9	0.95

Weight (kg)	67.4 ± 11.4	78.5 ± 13.9	0.004

Disease duration (days)	90 (12-720)	86 (24-210)	0.32

Morning stiffness (minutes)	100.7 ± 85.9	89.0 ± 52.2	0.69

Fatigue	26 (55.3%)	10 (76.9%)	0.28

Fever	10 (21.3%)	3 (30%)	0.81

Weight loss	16 (34%)	2 (15.4%)	0.34

Peripheral arthritis	13 (27.7%)	4 (30.8%)	0.89

Carpal tunnel syndrome	16 (34.0%)	2 (15.4%)	0.34

RS3PE	8 (17.0%)	2 (15.4%)	0.78

Tenosynovitis	4 (8.5%)	1 (7.7%)	0.64

ESR (mm/h)	63.8 ± 25.8	62.5 ± 22.4	0.88

CRP (mg/L)	30 (1.5-180)	30 (2.5-247)	0.96

**Figure 2:**
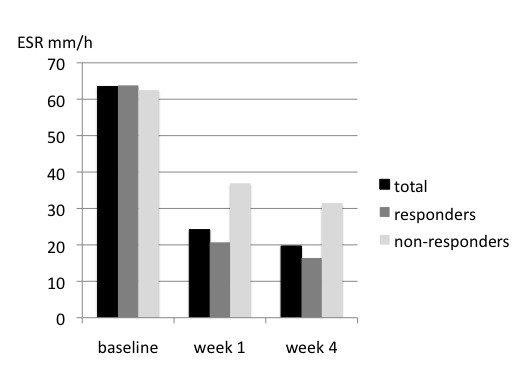
**Erythrocyte sedimentation rate (ESR) at baseline and at the week 1 and 4 control visits in the whole group of patients, in responders and non-responders**. There was a significant difference by repeated measures ANOVA between responders and non-responders (p = 0.017) and within groups (p < 0.001).

**Figure 3:**
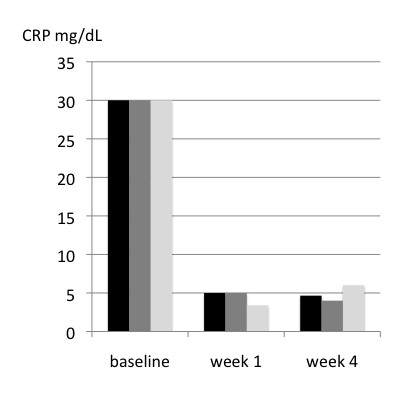
**C-reactive protein (CRP) at baseline and at the week 1 and 4 control visits in the whole group of patients, in responders and non-responders**. There was a significant difference by repeated measures ANOVA between responders and non-responders (p = 0.044) and within groups (p < 0.001).

The changes of ESR and CRP during the study period are reported in figure 2 and 3, respectively. There was a significant decrease of inflammatory parameters both in responders and non-responders. However, responders had a more pronounced decrease than non-responders.

Of the 13 non-responders, 7 still had PMR signs and symptoms, 5 has elevated ESR, and 3 elevated CRP, with only one patient showing both elevated CRP and ESR. Their dose of prednisone had been increased within one month from initiation of therapy between 2.5 and 12.5 mg/day with a resulting mean dose of 21.1 ± 3.2 mg/day. In this group, remission was reached after a mean interval of 41.2 ± 21.8 days. After dose adjustment, their mean prednisone dose per kg was 0.27 ± 0.06 mg. The median dose per kg for the whole group was 0.20 mg (range 0.13 mg-0.37 mg; 95% CI 0.19 mg-0.21 mg).

At the informal chart review performed at 6 months from study initiation, 14/60 (23.3%) of the patients had had one or more exacerbations. They occurred in 8/47 (17%) responders and in 4/13 (30.8%) non-responders (p = 0.48).

## Discussion

Our results support the hypothesis that a low initial dose of prednisone is sufficient to control PMR in the majority of patients. They also suggest that 0.20 mg per kg weight could be the adequate starting dose, although these data should be confirmed in prospective studies in which steroid dose is adjusted to body weight. It is possible that, due to the open design of the study, a placebo effect increased the response rate of our patients. However, since no comparison was made between different treatments, we think it could not have biased the results. Responders reached clinical remission in 6.6 days in average; most of them within 10 days from onset of therapy. This observation suggests that a close follow-up in the first days after diagnosis and treatment initiation is important to ensure that the patient is administered an adequate prednisone dosage.

There is only one comparison of two different dosages of GC in the literature [7]: PMR patients were randomly assigned to an initial regimen of 10 mg or 20 mg prednisolone and followed for two months. The patients on the low dose GC regimen had a higher incidence of relapses during the follow-up. The same incidence of giant cell arteritis was seen in both groups. No attempt was made to correlate efficacy with body weight in this study. Conversely, an initial prednisolone dose of 10 mg/day was felt adequate by Behn et al. [8], with only 8/67 (11.9%) PMR patients needing an increase in dosage. Another study used a standardized schedule of prednisone with a starting dose of 20 mg/day [9]. Twenty out of 27 patients (74%) reached remission with this regimen, a percentage similar to that obtained in our study with a much lower dose. This observation suggested that there is a subset of a quarter of PMR patients with steroid-resistant disease [9], regardless of the initial GC dose utilized. Other observational studies used initial GC mean doses comprised between 12.8 mg [10] and 22.8 mg [11]. However, due to lack of clinical information, it is impossible to derive from these papers how effective was GC in the initial period of treatment.

Disease activity, evaluated by clinical, laboratory, and ultrasonographic parameters, was not important to predict response to therapy. However, the power to test the predictive value of US was probably low because of the limited number of patients in whom the examination was performed. In univariate analysis, women showed a slightly better response to treatment than men. In contrast, previous data on PMR [12] and rheumatoid arthritis [13] have reported higher disease severity and lower rate of response to GC in women. In fact, when multivariate analysis was performed on our cases, the association of female sex to response appeared spurious, being related to the lower mean weight of women. The observation that the optimal starting dose depends on weight and not on disease activity, and is relatively low, may confirm the view that GC action in PMR is more of replacement type than anti-inflammatory [14]. The importance of body weight in the response to prednisone treatment is not surprising, in view of the fact that GC have a high volume of distribution and are highly lypophilic [15].

One of the limitations of this study is that clinical assessors could not be blinded to patient's weight. In addition, the design of the study limited the follow up to only one month. To partially overcome this last limitation, we performed an informal chart review to assess the presence of exacerbations within the first 6 months of treatment. Only 23% of the patients had one or more exacerbations with no differences between initial responders and non-responders. As a result, the rate of exacerbations was low in comparison with other studies using similar [7] or higher initial doses of GC [9,11] and response to a lower initial dose of prednisone was not correlated with a higher incidence of exacerbations. Although we cannot assume that a low GC starting dose necessarily corresponds to a low cumulative dosage, this is suggested by several observational studies [5].

Response to GC is one of the classification criteria for PMR in most studies [16], but the dose at which the drug should be administered to the effect has not been defined. An international group has recently addressed this problem by consensus, with rapid response to steroids defined as > 75% global response within 1 week to 15-20 mg daily of prednisone [17]. The BSR-BHPR guidelines suggest the same [2], but there is only type C evidence to support this view [18]. Our data could help standardize the optimal starting dose. They suggest that, in low-weight patients, this dose could be lower than that previously suggested. In fact 78.3% of our patients could benefit from a starting dose below 15 mg prednisone. This is in keeping with the EULAR recommendations to use the lowest possible GC dose in PMR [19]. The mean time interval needed to reach remission in our cohort of responsive patients was similar to that suggested by the panel of experts.

## Conclusions

In conclusion, in our experience low dose GC was effective in the majority of PMR patients and the main factor driving response to steroids in PMR was weight, a finding that could help to manage the clinical care of PMR patients and design prospective studies of treatment.

## List of Abbreviations

PMR: Polymyalgia rheumatica; GC: Glucocorticoids; RS3PE: Remitting seronegative symmetric synovitis with pitting edema; US: Ultrasonography; ESR: Erythrocyte sedimentation rate; CRP: C-reactive protein; RF: IgM rheumatoid factor

## Competing interests

The authors declare that they have no competing interests.

## Authors' contributions

MAC and RC designed the study; MAC, RC and MP followed clinically the patient's cohort; MP performed US; MAC performed the statistical analysis; MAC, RC and CM drafted the manuscript and revised it critically; all the authors read the final manuscript and gave their approval.

## Pre-publication history

The pre-publication history for this paper can be accessed here:

http://www.biomedcentral.com/1471-2474/12/94/prepub
